# Children's Evaluations of Empathizers

**DOI:** 10.1111/cdev.14242

**Published:** 2025-04-04

**Authors:** Alexis S. Smith‐Flores, Gabriel J. Bonamy, Lindsey J. Powell

**Affiliations:** ^1^ Department of Psychology University of California La Jolla California USA

**Keywords:** empathy, prosocial behavior, social evaluation

## Abstract

Children's evaluations of empathizers were examined using vignette‐based tasks (*N* = 159 4‐ to 7‐year‐old U.S. children, 82 girls, 52% White) between March 2023 and March 2024. Children typically evaluated empathizers positively compared to less empathic others. They rated empathic responses as more appropriate, selected empathizers as nicer, and inferred more positive relationships between empathizers and the targets of empathy. However, when empathy was contrasted with helping behavior, or directed toward an immoral actor, evaluations of empathy were more negative. Older children weighed helping and empathy more equally and shifted their evaluations more when considering responses to immoral acts. These results show children use empathy in their social evaluations, and contextual influences on these evaluations strengthen with age.

People judge others not just by their actions but also by the intentions and motives associated with those actions (Barrett and Saxe 2021; Carlson et al. 2022; Cushman 2008; Uhlmann et al. 2015). This tendency to appeal to underlying mental states during social evaluation develops by early childhood (Killen et al. 2011; Yuill and Perner 1988). For example, if 5‐year‐old children are told that one boy—let's call him Anthony—tried to push another child over but missed, and that another boy, Cameron, tripped and pushed someone over by accident, they rate Anthony as both naughtier and more worthy of punishment than Cameron, even though Cameron caused harm and Anthony did not (Cushman et al. 2013).

Here, we ask if children also evaluate others on the basis of another type of internal, mental state: vicarious emotions, or emotions arising in response to others' experiences. Vicarious emotions can be broadly empathic or compassionate (i.e., congruent with the valence of the other person's experience and likely emotion) or they can be broadly counter‐empathic (i.e., incongruent with the valence of the other person's experience and likely emotion). Say Michael was watching when Cameron accidentally pushed his classmate over. Michael may either feel sad for his fallen classmate's pain or, alternatively, feel happy about the classmate's misfortune at being accidentally pushed down. Do such empathic or counter‐empathic emotions influence children's evaluations of Michael? If so, this could suggest that, like adults, children take vicarious emotions as evidence of underlying traits, motives, or moral character (Carlson et al. 2022; Fiske et al. 2007; Goodwin et al. 2014).

Before proceeding, we note that although some definitions of affective empathy restrict it to cases in which the empathizer exactly matches an emotion expressed by a target person (e.g., Preston and Waal de 2002), here we adopt a broader definition that also encompasses “empathic care” that is, a feeling of warmth toward others and concern for their welfare (Ashar et al. 2017; Batson 2011; Depow et al. 2021). We use counter‐empathy to encompass both “schadenfreude”—happiness about someone else's misfortune—and “gluckschmerz”—sadness or pain at someone else's happiness (Hudson et al. 2019; Yamada et al. 2011).

## 1 Existing Evidence

Adults do judge others on the basis of observed vicarious emotions, typically favoring empathizers. For example, doctors and salespeople who empathize with their patients and clients are viewed as more trustworthy (Aggarwal et al. 2005; Kim et al. 2004). Adults say that empathy provides information about both a person's moral character and their warmth (Barasch et al. 2014; Goodwin et al. 2014). When told vignettes about people who responded in empathic versus non‐empathic ways to others' everyday struggles, adults rated the empathizing responder as more respectable, likable, and warm (Wang and Todd 2021).

There is also some evidence that children use vicarious emotions in social evaluation. Several studies find that young children positively evaluate puppets who overtly express concern through comforting, relative to puppets who ignore another's suffering or approach the sufferer to laugh at them (Geraci et al. 2021; Paulus et al. 2020). Children also sometimes admonished or protested against the puppets who laughed at others' pain (Paulus et al. 2020). Older children also endorse comforting as desirable or obligatory, especially when the target is highly distressed (Tavassoli et al. 2023).

Other research finds that children, and also infants in some circumstances, can use information about social relationships to guide expectations about vicarious emotions. Infants and young children expect friends or ingroup members, but not rivals or outgroup members, to be happy for one another's success or good fortune (Smith‐Flores et al. 2023, 2024; Tompkins et al. 2024). Older children, like adults, also expect less empathic concern from a rival or outgroup member than from a friend or ingroup member, though younger children expect empathic concern from all observers regardless of affiliation (Smith‐Flores et al. 2023; Tompkins et al. 2024). Across the ages tested (4–7 years), children also used observations of empathy and counter‐empathy to infer positive and negative social relationships, respectively (Smith‐Flores et al. 2023). This body of findings suggests children perceive a link between vicarious emotions and interpersonal care.

## 2 Current Research

Here we seek to extend the existing research in several ways. First, we investigate if children evaluate others based on their vicarious emotional responses alone, in the absence of overt prosocial or antisocial behavior toward the subject of those emotions, such as comforting or mocking. For a number of reasons, including the fact that vicarious emotions can be expressed even when the subject is absent, information about these emotions is likely to be more prevalent than observation of overt comforting or mocking behaviors. Thus, assessing children's judgments based directly on vicarious emotions will help determine how broad a role they may play in children's overall social evaluations and, more generally, test the extent to which children rely on mental state information in their judgments about others. To keep the focus on vicarious emotions, we also tried to limit overt similarity or imitation between targets and empathizers, as these dimensions are known to elicit positive evaluations from children (Fawcett and Markson 2010; Pesowski et al. 2023; Roberts et al. 2017). We did this by omitting direct description or illustration of the targets' emotions, which could be readily inferred from information provided about their goals and outcomes.

Second, in addition to asking *if* children use vicarious emotions in their social evaluations, we also aim to understand the social cognitive basis of such evaluations. One possibility is that children have learned that expressions of empathy are normative or “nice,” while expressions of counter‐empathy—especially in the context of others' suffering—are unacceptable or “mean.” On this view, judgments of vicarious emotions would rely primarily on norm psychology (Paulus et al. 2020). Relationship inferences, too, could rely on the heuristic that people are nice to their friends.

An alternative possibility, not mutually exclusive with the normativity account, is that children employ intuitive psychology when reasoning about vicarious emotions. Young children are able to reason about emotions as the outcome of an appraisal process and to infer the beliefs and desires that likely gave rise to them (Doan et al. 2023; Wellman et al. 2000; Wu et al. 2017). Using this intuitive psychology, children may infer that vicarious emotions result from how the emoter values the subject's welfare. Empathic emotions would arise from an observer's desire for positive, welfare‐enhancing experiences for the subject, while counter‐empathic emotions would arise from an observer's desire for the subject to experience suffering or otherwise reduced welfare (Hudson et al. 2019; Smith‐Flores and Powell 2023; Wondra and Ellsworth 2015). The inference of these prosocial or antisocial values would then serve as a basis for social evaluation. However, this latter account also makes the prediction that children may not always evaluate empathy positively: When empathy is directed toward a subject pursuing an antisocial or immoral goal, it would indicate the empathizer's support for that goal, potentially leading to a negative social evaluation of the empathizer. This would also be consistent with the finding that adults negatively evaluate those who empathize with White supremacists relative to those who condemn them (Wang and Todd 2021).

Finally, there is a question of how empathy is considered in social evaluations in combination with other evidence of a person's character. One factor may be the perceived cost of empathy compared to other social behaviors, such as helping. People do not necessarily perceive empathy as cost‐free. Adults have been found to go out of their way to avoid bids for empathy because of added costs: monetary (Andreoni et al. 2017; Cameron and Payne 2011; Pancer et al. 1979), psychological (Cameron et al. 2016; Manczak et al. 2016), or cognitive (Cameron et al. 2019), for example. Children also rate comforting as costly (Tavassoli et al. 2023). Nonetheless, children might still view empathy as *less* costly, or less effective, than an action‐based prosocial behavior, such as helping. Asking children to make relative evaluations of people who empathize versus those who help will provide insight into the relative value they place on these two responses.

We addressed these three aims in two studies that presented children with vignettes about target characters who experienced some positive or negative outcome, and observers who witnessed those outcomes and then had vicarious emotional responses that were empathic, neutral, or counter‐empathic. Across the two studies, children made judgments about the appropriateness, niceness, and relationships of the vicariously responding observers. In addition to varying the nature of the vicarious responses, we also varied whether these responses were contrasted with helpful behavior and whether the targets of the vicarious emotions had morally neutral vs. immoral goals. We tested children between the ages of 4 and 7, anticipating that we might observe increasingly nuanced reasoning with age, as both children's reasoning about vicarious emotion and their consideration of mental states in social evaluation have been shown to change within this developmental range (Cushman et al. 2013; Smith‐Flores et al. 2023; Tompkins et al. 2024). Overall, we hoped to shed light on the development of children's emotion‐based social evaluations.

## 3 Study 1

In the first study, we investigated how children evaluated the appropriateness, niceness, and social relationships of story characters who expressed empathic, neutral, or counter‐empathic responses to a target character's positive or negative outcome. This set of questions was designed to provide insight into how children evaluate the act of empathizing or counter‐empathizing, as well as how they believe that act reflects on the emoter's character and relationships. In order to accommodate this expansive space of evaluations, each story featured two emoters who displayed contrasting emotional responses to the target's outcome. Participants rated the appropriateness of each emoter's response and made forced choice judgments about which emoter was nicer and better friends with the target. In two of the stories, the emoting characters were also described as either offering or not offering help to the target character.

Our primary hypotheses were that children would rate empathy as more appropriate (i.e., more “okay”), nicer, and more indicative of friendship, compared to both counter‐empathy and neutral responses. We also had two exploratory aims. The first was to investigate if children would find counter‐empathy less appropriate, less nice, and more indicative of a negative relationship when compared to a neutral emotional response. If children believe counter‐empathy reflects antisocial behavioral tendencies or motivations, then they may judge counter‐empathizers as less nice than both empathizers and neutral responders. However, if children's judgments about empathizers versus counter‐empathizers are driven primarily by children's positive assessments of empathy, or if neutral responses are seen as negative (as in Paulus et al. 2020), then children may evaluate the counter‐empathizer and neutral character similarly.

The second exploratory aim was to investigate how children's evaluations of empathy compare to their evaluations of helping. In stories that featured both, one character was associated with empathy but not helping, and another with helping but not empathy. If children view empathy as a normative prosocial behavior with equal weight to helping, then they may evaluate characters who engage in these behaviors similarly and be at chance when selecting whether a helper or an empathizer is nicer. However, if they view either empathy (as an internal mental state reflecting motivation) or helping (as a more costly or effective response) as stronger evidence of prosociality, then we should find children more frequently select one of the character types than the other when asked who is nicer.

These hypotheses, our materials, data collection procedure, and analysis plan were preregistered on OSF: https://osf.io/b6qwg/.

### 3.1 Methods

#### 3.1.1 Participants

Seventy‐two children, aged 4–7 years old, participated at a children's science museum in Southern California (*M*_age_ = 6.06 years, SD_age_ = 1.07 years, 37 girls). Sample size was determined based on a previous study that investigated children's relationship ratings based on vicarious emotions (Smith‐Flores et al. 2023). A power analysis simulation based on those data suggests we would have 100% power to detect rating differences with a similar effect size of Cramer's *V* = 0.91, even with a substantially smaller sample, though we retained the current sample size in case the effect of emotion on appropriateness ratings was somewhat smaller (Green and MacLeod 2016). An additional four children were tested but were excluded from the final analysis due to preregistered criteria, including failing the warm‐up question (1), experimenter error (1), and being outside the preregistered age range (2).

Caregivers provided informed consent and completed an optional demographic form. Thirty‐seven children were identified by their caregivers as White, 9 as Asian, 2 as American Indian or Alaskan Native, 2 as Black or African American, 15 children were identified as belonging to two or more races, and 7 caregivers declined to answer. Nineteen children were identified as Hispanic or Latinx and 3 declined to answer. Sixty children came from families where at least one caregiver had a college degree or higher, 7 had at least one caregiver who completed some college, 2 had at least one caregiver who had a high school diploma or GED, and 3 caregivers declined to answer. Participants received a small gift (i.e., temporary tattoos, stickers, or toys) as compensation for their participation. This study and its procedure were approved by the university's institutional review board. Data collection occurred between March 2023 and August 2023.

#### 3.1.2 Materials and Procedure

##### 3.1.2.1 Warm Up

**FIGURE 1:**
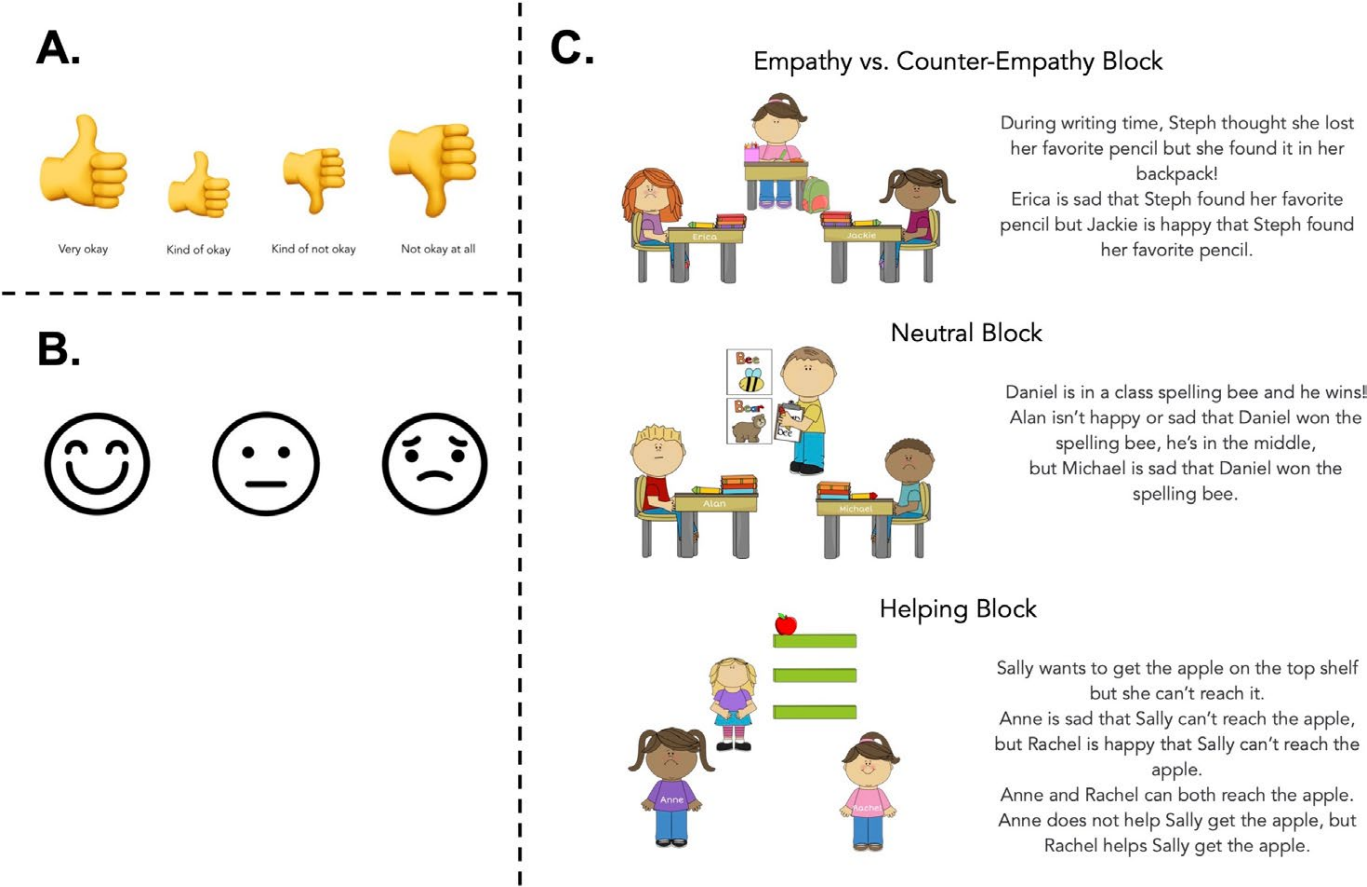
(A) Children in Study 1 were asked to infer how okay it was for each emoter to emote the way they did using a 4‐point scale ranging from “very okay” to “not okay at all.” (B) For the manipulation check, children were asked to repeat how each emoter feels using a 3‐point scale depicting a happy face, a sad face, and a neutral face, referred to as being “in the middle” of happy and sad. (C) Example stimuli from the Empathy Block, Neutral Block, and Helping Block.

The researcher first introduced a 4‐point scale, ranging from “very okay” to “not okay at all,” accompanied by a thumbs up or thumbs down icon which varied in size depending on extremity of the rating (Figure 1A). The researcher asked participants how okay it would be if they were given stickers (with the expectation that they would say “very okay” or “kind of okay”), and how okay it would be if all of their stickers were stolen (with the expectation that they would say “not okay at all” or “kind of not okay”). Participants responded verbally or by pointing to the scale. The researcher then introduced a 3‐point scale depicting a smiley face, a sad face, and a neutral face (Figure 1B). This scale was used to familiarize participants with the concept of a child responding in an emotionally neutral way, or being “in the middle”, as they were told, and was used to administer manipulation checks following each story, when children were asked to recall how the emoting characters felt: happy, sad, or in the middle, meaning that they are neither happy nor sad.

##### 3.1.2.2 Empathy vs. Counter‐Empathy Block

The first block of trials contained two stories. In both stories, one emoter responded empathically to the target's experience, while the other emoter responded counter‐empathically (Figure 1C). In one story, the target's outcome was positive, while in the other story it was negative (positive outcome example: “During writing time, Steph thought she lost her favorite pencil, but she found it in her backpack!”). Participants were told about the emoters' reactions to the target character's outcome (e.g., “Erica is sad that Steph found her favorite pencil, but Jackie is happy that Steph found her favorite pencil.”). Story order and whether the empathic emoter was described first or second were orthogonally counterbalanced across participants. In the positive outcome story, the empathic emoter was happy and the counter‐empathic emoter was sad; in the negative outcome story, the empathic emoter was sad and the counter‐empathic emoter was happy. After each story, participants were asked the manipulation check question followed by the test questions.

##### 3.1.2.3 Comparison‐to‐Neutral Block

The next block followed the same format, except that one of the emoters in each story reacted neutrally, described as feeling neither happy nor sad about the target's outcome but rather “in the middle.” Both stories featured a positive outcome for the target character. One story paired the neutral responder with an empathic responder and the other with a counter‐empathic responder. Whether the neutral responder was introduced first was counterbalanced across stories and the order of stories was counterbalanced across participants. Each story was followed by the manipulation check and test questions.

##### 3.1.2.4 Helping vs. Empathy Block

In the final block, the target character in each of the two stories was unable to complete a goal (e.g., to reach an apple on a high shelf). One of the emoting characters was described as feeling counter‐empathic happiness at the target's failure, but also acting to help accomplish the target character's task. The other emoter was described as feeling empathic sadness for the target, but not offering help. Whether the unhelpful empathizer was introduced first or second was counterbalanced across stories, and the order of stories was counterbalanced across participants. The questions following each of these stories were the same, with the addition of a second manipulation check which determined if participants could recall which character helped.

##### 3.1.2.5 Manipulation Checks

After each story, participants were asked to use the emotion scale to recall how the two emoters felt following the target's outcome (e.g., “Remind me, how does Erica feel? And how does Jackie feel?”). Participants had to point to a face or verbally respond to the researcher. Participants who did not answer correctly for both characters heard the story again and were asked once more. Participants who did not provide both correct answers after the second retelling for any story were excluded from that story's analysis (*n* = 3). All participants who received a second prompt answered and were included in the final analysis. In the Helping Block, we also asked participants which of the two emoters helped the target character (e.g., “Remind me, who helped Sally, Anne or Rachel?”) Participants who did not answer correctly heard the story again and were asked once more. Participants who did not provide a correct answer after the second retelling for the story were excluded from that story's analysis (*n* = 0).

##### 3.1.2.6 Dependent Variables

For all three blocks, participants were asked four test questions about each story across three question types: an appropriateness evaluation for each emoter, a relative social evaluation of niceness, and a relative relationship inference. The appropriateness evaluations were always done first, while the order for the social evaluation and relationship inference was counterbalanced across participants. For all test questions, participants who did not provide an answer after an initial prompt were prompted a second time before moving on to the next story. No participants were excluded for this reason.

###### 3.1.2.6.1 Appropriateness Evaluation

Participants were asked to evaluate the appropriateness of each character's response using the “okay” scale from the warm‐up (e.g., “How okay was it for Erica to feel sad that Steph found her favorite pencil? Very okay, kind of okay, kind of not okay, not okay at all?”).

###### 3.1.2.6.2 Social Evaluation

Participants were asked to indicate which of the two emoting characters is nicer in a binary choice question (e.g., “Who is nicer, Erica or Jackie?”). We coded which of the two emoters participants chose for each comparison type (e.g., empathizer vs. counter‐empathizer or empathizer vs. neutral).

###### 3.1.2.6.3 Relationship Inference

Participants were also asked to infer which of the two characters were friends with the target character in a binary choice question (e.g., “Who is Kyle friends with, Erica or Jackie?”). We again coded which of the two emoters participants chose for each comparison type.

#### 3.1.3 Description of Analyses

##### 3.1.3.1 Appropriateness Evaluations

To analyze data from the four “okay” questions in the Empathy versus Counter‐empathy block (two per story), we preregistered nested comparisons of mixed effects linear models. Response type (empathy vs. counter‐empathy), outcome type (positive vs. negative), and the interaction between response type and outcome type were included in the full model as fixed effects. Response type and outcome type were dummy‐coded and mean‐centered. We planned to include participant ID as a random effect. We also conducted non‐preregistered, exploratory analyses of potential developmental change by adding participant age and its two‐and three‐way interactions with response type and outcome to the full model and comparing it to alternative models with each age term removed.

For the Comparison‐to‐Neutral block, we preregistered separate analyses of the okayness ratings for the empathizer vs. neutral and counter‐empathizer versus neutral stories. For each story type, we used Wilcoxon signed‐rank tests to compare ratings for the two emoters.

In the Helping versus Empathy block, we conducted preregistered analyses of all okayness ratings from the two stories using nested comparisons of mixed effects linear models. Response type (unhelpful empathy vs. helpful counter‐empathy), story type (apple vs. book), and the interaction between response type and story type were included in the full model as fixed effects. Response type and story type were dummy‐coded and mean‐centered. We planned to include participant ID as a random effect. Again, we ran additional exploratory comparisons including participant age and interactions with age in the full model.

##### 3.1.3.2 Social Evaluation

To test our hypotheses that children would rate empathizers as nicer than counter‐empathizers or neutral responders, and to explore their relative niceness evaluations in the Comparison‐to‐Neutral and Empathy versus Helping blocks, we conducted separate binomial tests for each of the six stories. For the social evaluation and relationship inference questions from the Empathy versus Counter‐empathy and Empathy versus Helping blocks, we initially preregistered logistic regression analyses employing models similar to the one used for the appropriateness ratings. However, the planned models were inappropriate for these data given that participants chose between the two emoters in the social evaluation and relationship inference question, rather than giving separate ratings for each. We thus deviated from the preregistration in our analyses of these data.

##### 3.1.3.3 Relationship Inference

In the Empathy versus Counter‐empathy block, we expected to replicate the finding that children infer closer relationships following empathic responses than following counter‐empathic responses (Smith‐Flores et al. 2023). The Comparison‐to‐Neutral block can extend these findings by separately testing if children think empathic responders are more likely and counter‐empathic responders are less likely to be friends with the target than a neutral character. The previous research also did not ask about conflicting prosociality cues to relationships. It may be that children find helping to be a more salient cue to friendship when it conflicts with empathy. To analyze data from the relationship inference question, we compared participants' choice between the two emoters using separate binomial tests for each story.

### 3.2 Coding

All responses were coded by the second researcher at the time of the experiment. An additional coder coded 20% of participants from video recordings to ensure reliability. Agreement between coders was 100%.

### 3.3 Results

#### 3.3.1 Empathy vs. Counter‐Empathy Block

##### 3.3.1.1 Appropriateness Evaluations

We fit our preregistered mixed‐effect linear regression model of children's appropriateness evaluations to the data from the Empathy versus Counter‐Empathy block. However, the participant‐level random effect variance was close to zero, causing a convergence issue. So, we removed the term for the random effect of the participant, resulting in a full model as follows:

rating~response type+outcome type+response type:outcome type

Using nested model comparisons, we then contrasted this full model with models that excluded each factor individually to test how much the excluded factor contributed to explaining the variance in children's evaluations.

There was a main effect of response type, *F*(1, 284) = 73.586, *p* < 0.001. Children rated empathic responses (*M* = 3.083, SEM = 0.101) as more okay than counter‐empathic responses (M = 1.924, SEM = 0.094). Post hoc one‐sample t‐tests showed that children's mean ratings of counter‐empathic responses across the two stories were significantly below the midpoint of the scale, which separated “okay” from “not okay” ratings (*t*(71) = −3.90, *p* < 0.001), while mean ratings of the empathic responses were significantly above the midpoint (*t*(71) = 5.41, *p* < 0.001). There was also a main effect of outcome type, *F*(1, 284) = 5.829, *p* = 0.016. Children rated responses to negative outcomes (*M* = 2.340, SEM = 0.108) as less okay than responses to positive outcomes (*M* = 2.667, SEM = 0.108).

**FIGURE 2:**
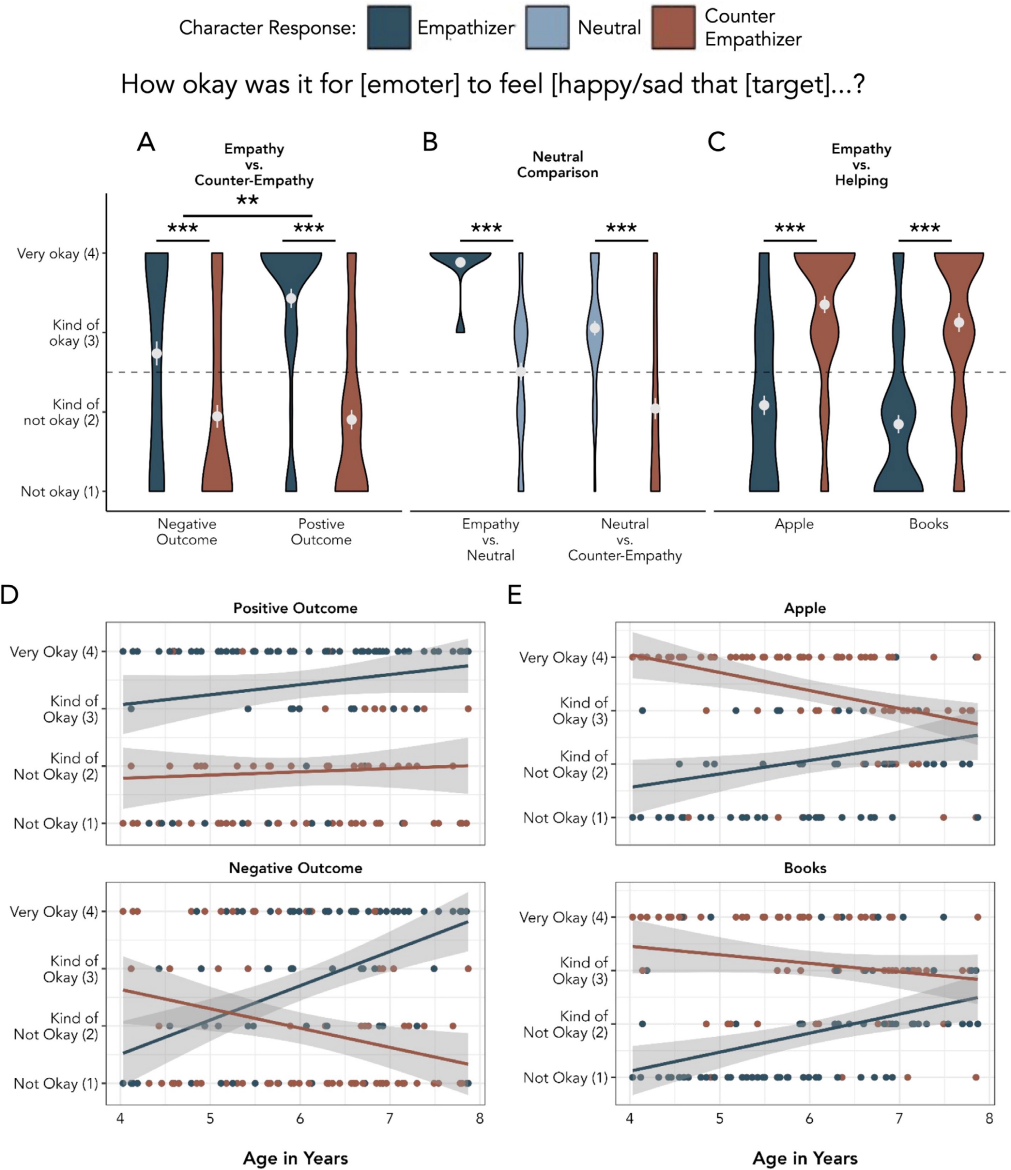
Results from Study 1 Appropriateness Evaluations. (A–C) Children's appropriateness evaluations for each block. Means and standard errors are plotted in white. The dashed line represents the mid‐point of the scale (2.5). (D) Children's appropriateness evaluations across age for the Empathy versus Counter‐Empathy Block. Each dot represents individual children's responses for each story. The gray shaded area represents 95% confidence intervals. (E) Children's appropriateness evaluations across age for the Helping Block where empathy and helping behaviors were contrasted against one another. **p < 0.01, ***p < 0.001.

Finally, there was also a significant interaction between empathy type and outcome type, *F*(1, 284) = 7.412, *p* = 0.007 (Figure 2A). We used post hoc paired sample t‐tests with Bonferroni adjustments (α = 0.0125, 0.05 divided by 4 tests) to interrogate this interaction. We found that children rated empathic responses as more okay than counter‐empathic responses following both positive and negative outcomes (*t*'s > 4.141, *p*'s < 0.001). Additionally, children's ratings of the okayness of counter‐empathic responses did not differ significantly across positive (*M* = 1.903, SEM = 0.124) and negative outcomes (*M* = 1.944, SEM = 0.142), *t*(284) = 0.218, *p* = 0.828, 95% CI [−0.335, 0.418], *d* = 0.037. However, children rated empathic responses as more okay following positive outcomes (*M* = 3.431, SEM = 0.122) than negative outcomes (*M* = 2.736, SEM = 0.151), *t*(284) = −3.632, *p* < 0.001 [−1.071, −0.318], *d* = −0.605. In other words, children rated empathic happiness as more “okay” than empathic sadness. One possible explanation for this is that children, especially younger children, may have difficulty reporting that feeling sad is “okay,” even if sadness is the most appropriate emotional response.

In our exploratory age analyses, a nested model comparison found a significant three‐way interaction between age, empathy type, and outcome type, *F*(1, 280) = 11.398, *p* < 0.001 (Figure 2D). Post hoc comparisons showed that relative ratings of empathy and counter‐empathy following positive outcomes did not change with age, estimate_diff_ = 0.119, *t*(280) = 0.697, *p* = 0.487, [−0.217, 0.456], *d* = 0.109. However, relative ratings for empathic and counter‐empathic responses following negative outcomes did interact with age, estimate_diff_ = 0.935, *t*(280) = 5.471, *p* < 0.001, [0.599, 1.272], *d* = 0.858. As children got older, they were more likely to rate empathic sadness as “okay” and counter‐empathic happiness as “not okay”.

##### 3.3.1.2 Social Evaluation

**FIGURE 3:**
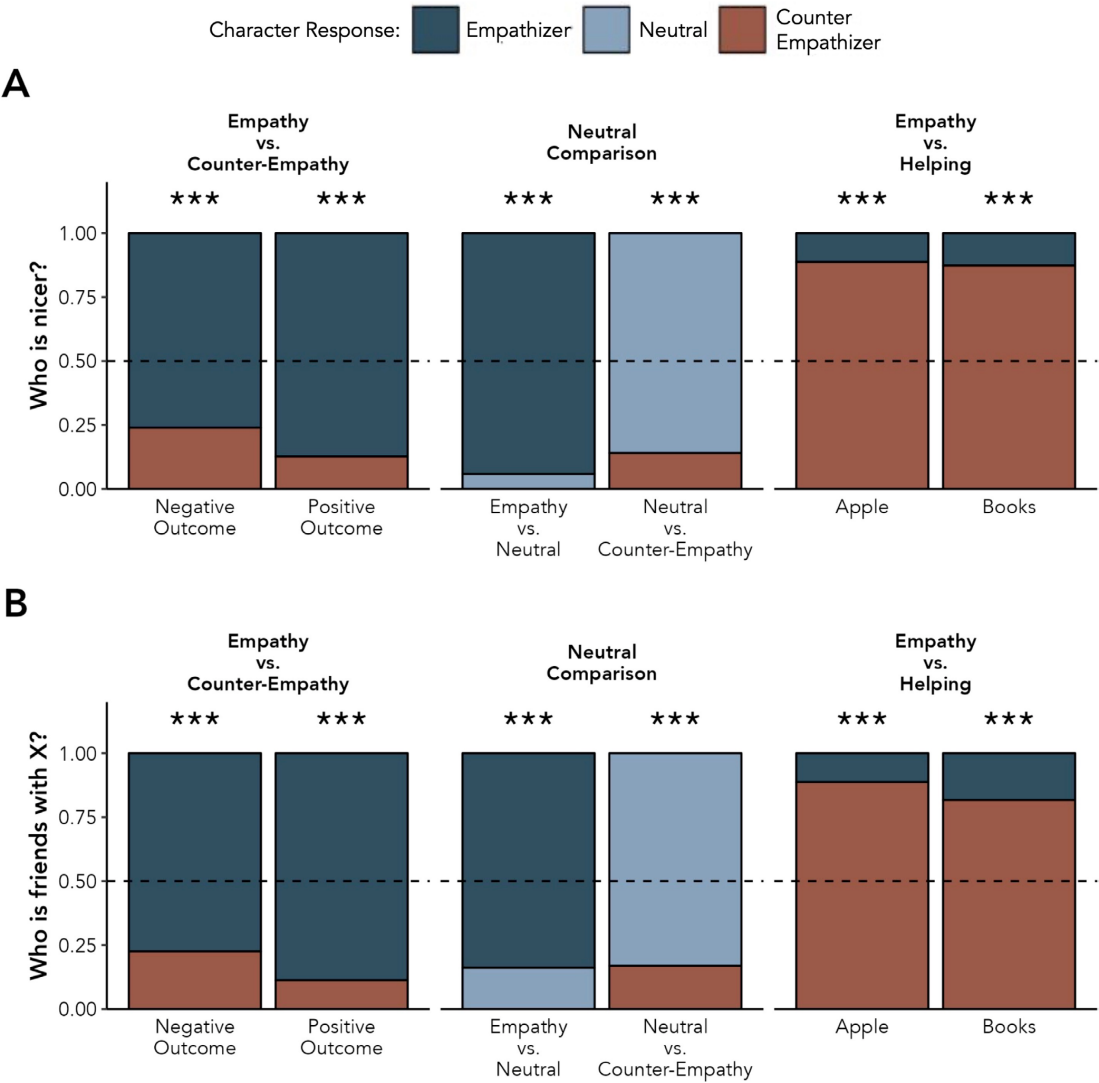
Children's responses to the social evaluation and relationship question in Study 1. The y‐axis reflects the proportion of children's responses to the social evaluation (A) and the relationship inference (B). The dashed line represents chance performance. ***p < 0.001.

Participants selected the empathizer as nicer than the counter‐empathizer more often than would be expected by chance following both stories, positive outcome: *N* = 62/72, 86.1%, *p* < 0.001, 95% CI [0.759, 0.931]; negative outcome: *N* = 54/72, 75%, *p* < 0.001 [0.634, 0.845] (Figure 3A).

##### 3.3.1.3 Relationship Inferences

When asked to select which emoter was friends with the target, participants selected the empathizer at above chance rates, positive outcome: *N* = 63/72, 87.5%, *p* < 0.001, 95% CI [0.776, 0.941]; negative outcome: 55/72, 76.4%, *p* < 0.001, 95% CI [0.649, 0.856] (Figure 3B).

#### 3.3.2 Comparison‐to‐Neutral Block

##### 3.3.2.1 Appropriateness Ratings

Wilcoxon signed‐rank tests showed that, as predicted, children rated an empathic response as more “okay” (*M* = 3.884, SEM = 0.039) than a neutral response (*M* = 2.507, SEM = 0.110), *V* = 1918, *p* < 0.001, *r* = 0.839. Children also rated a neutral response as more “okay” (*M* = 3.056, SEM = 0.092) than a counter‐empathic response (*M* = 2.042, SEM = 0.134), *V* = 282, *p* < 0.001, *r* = 0.627 (Figure 2B). Post hoc one‐sample t‐tests again showed that ratings of empathic responses were significantly above the midpoint separating “okay” from “not okay” ratings (*t*(68) = 35.65, *p* < 0.001), while ratings of counter‐empathic responses were significantly below the midpoint (*t*(70) = −3.423, *p* = 0.001). Ratings of the neutral responses did not differ significantly from the midpoint when contrasted with an empathic response (*t*(68) = 0.066, *p* = 0.948), but were significantly above the midpoint when contrasted with a counter‐empathic response (*t*(70) = 6.068, *p* < 0.001).

##### 3.3.2.2 Social Evaluation

Following the story about an empathizer and a neutral responder, 64 of 68 participants (94.1%) selected the empathizer as nicer than the neutral character, *p* < 0.001, 95% CI [0.856, 0.984]. When the neutral character was compared to the counter‐empathizer, 61 of 71 participants (85.9%) selected the neutral character as nicer than the counter‐empathizer, *p* < 0.001, 95% CI [0.756, 0.930] (Figure 3A).

##### 3.3.2.3 Relationship Inferences

Fifty‐seven of 68 participants (83.8%) selected the empathizer over the neutral character as being friends with the target character, *p* < 0.001, 95% CI [0.729, 0.916]. When the neutral character was compared to the counter‐empathizer, 59 of 71 participants (83.1%) selected the neutral character as being friends with the target character instead of the counter‐empathizer, *p* < 0.001, [0.723, 0.910] (Figure 3B).

#### 3.3.3 Helping vs. Empathy Block

##### 3.3.3.1 Appropriateness Ratings

When we fit mixed effects linear models of children's appropriateness ratings from the final block, participant‐level random effect variance was close to zero, so we removed this term to avoid issues with model convergence. The resulting full model was as follows:

rating~response type+trial+response type:trial

There was a main effect of response type, *F*(1, 280) = 120.142, *p* < 0.001—children rated counter‐empathic but helpful responses as more “okay” (*M* = 3.239, SEM = 0.081) than empathic but unhelpful responses (*M* = 1.965, SEM = 0.084). Mean ratings of the helpful counter‐empathizer were significantly above the midpoint (*t*(71) = 7.76, *p* < 0.001), while mean ratings of the unhelpful empathizer were significantly below the midpoint (*t*(71) = −5.91, *p* < 0.001).

There was also a main effect of story, *F*(1, 280) = 3.994, *p* = 0.047—children rated all responses as slightly but significantly more “okay” in the story featuring a character who could not reach an apple (*M* = 2.718, SEM = 0.097) than in the story about a character who needed help picking up books (*M* = 2.486, SEM = 0.099). Finally, there was not a significant interaction between response type and story, *F*(1, 280) = 0.004, *p* = 0.952. These results suggest that helping carries more weight for children than empathy when they are considering whether a response to others' needs is appropriate (Figure 2C).

When we added age and the associated interactions as fixed effects to the full model described above, a nested model comparison found a significant two‐way interaction between age and response type, *F*(1, 276) = 28.006, *p* < 0.001. Relative ratings of empathic, non‐helpful responses and counter‐empathic, helpful responses changed with age, estimate_diff_ = 0.552, *t*(276) = 5.292, *p* < 0.001, CI [0.347, 0.758], *d* = 0.589. Older children's evaluations of unhelpful empathizers and helpful counter‐empathizers became more similar (Figure 2E).

##### 3.3.3.2 Social Evaluation

We conducted separate two‐tailed binomial tests for each trial (apple vs. book story). For both stories, we found that children were significantly less likely to select the empathic but unhelpful character as nicer (books story: *N* = 9/71, 12.7%, *p* < 0.001, [0.060, 0.227]; apple story: *N* = 8/71, 11.3%, *p* < 0.001, 95% CI [0.050, 0.210]) (Figure 3A).

##### 3.3.3.3 Relationship Inferences

In both stories, children were significantly more likely to say that the counter‐empathic but helpful character was better friends with the target than the empathic but unhelpful character (book story: *N* = 13/71 selected the empathic, unhelpful character, 18.3%, *p* < 0.001, 95% CI [0.101, 0.293]; apple story: *N* = 8/71 selected the empathic, unhelpful character, 11.3%, *p* < 0.001, [0.050, 0.210]) (Figure 3B).

### 3.4 Discussion

The results of Study 1 indicate that 4‐ to 7‐year‐old children evaluate empathic others more positively than counter‐empathic others. Moreover, evaluations of both empathy and counter‐empathy differed from children's evaluations of neutral emotional responses to others' experiences, in opposite directions. Participants rated empathic responses as more appropriate than both neutral and counter‐empathic responses, and selected empathizing characters as nicer than both neutral and counter‐empathizing characters. Conversely, children rated counter‐empathic responses as less appropriate than neutral responses and selected neutral characters as nicer than counter‐empathic ones. We also extended previous research on children's relationship inferences from vicarious emotions, finding that children not only believe that the targets of these emotions are closer friends with empathizers than counter‐empathizers, but they also use empathy and counter‐empathy to infer closer or more distant relationships relative to a neutral responder as well. This overall pattern of findings cannot be explained by imitation between the emoter and target, as we did not depict the target's response, nor simple endorsement of the emotion that “fits” the situation, as children distinguished neutral and counter‐empathic responses. The results are consistent with previous evidence of children's understanding that negative social relationships can lead to counter‐empathic emotions in situations like these (Smith‐Flores et al. 2023). Together, these findings support the hypothesis that from at least age 4 children use others' vicarious emotional responses, absent any overt behavior or expressions of these responses, as a basis for social evaluation, including both more positive evaluations of empathizers and more negative evaluations of counter‐empathizers, relative to a neutral responder.

A caveat to these findings is that, following a negative outcome, younger children were more likely than older children to rate empathic sadness as “not okay” and counter‐empathic happiness as “kind of” or “very okay.” Although we did not observe a similar age difference in ratings of empathy and counter‐empathy for positive outcomes, this could reflect children placing an increasing value on empathy with age. However, given that young children expect others to feel empathic concern (Smith‐Flores et al. 2023), and that other work has found 3‐year‐old children more positively rate individuals who express that concern via comforting compared to individuals who overtly express schadenfreude (Paulus et al. 2020), we suspect this developmental change is related to the difficulty of the question. Younger children's ratings may have been influenced by how okay they thought it was for a person to be sad in general. Given the ambiguity of interpreting responses to this item and these age‐related differences, we chose to forgo the appropriateness evaluation in our subsequent study.

The final block of trials found that children's positive empathy evaluations are not fixed, but rather they depend on the context of the empathy. Overall, children selected the counter‐empathic but helpful character as nicer and more likely to be friends with the target character instead of the empathic but unhelpful character. However, younger children weigh helping or more salient prosocial behaviors more positively than older children, who appear to consider both factors in their evaluations.

## 4 Study 2

Study 2 aimed to further understand the basis for children's evaluation of empathizers and counter‐empathizers by asking if those evaluations are impacted by the moral status of the actor and outcome that are the targets of the (counter‐)empathizer's vicarious emotions. Four of the six stories involved target goals typically judged to violate moral or conventional principles, such as physical harm, stealing, destruction of property, and rule‐breaking. In all stories, the target was described as wanting to cause the immoral outcome and then succeeding at doing so. Children in our age range typically judge that the actor in such situations is happy about their actions and outcome (i.e., the “happy victimizer” effect; Keller et al. 2003; Yuill and Perner 1988). The remaining two stories involved target goals that did not violate a moral or conventional principle, such as completing a puzzle and playing on the monkey bars. In each story, there was just one emoter, who responded to the target actor's success with either empathic happiness or counter‐empathic sadness. Participants rated how nice the emoter is and rated the closeness of the emoter's relationship to the target.

If children base their evaluations of vicarious emotions solely on whether or not the emoter empathizes with their target, then participants should rate the empathizer as both nicer and better friends with the target across all stories, regardless of the moral status of the target's goal. However, if children's evaluations of empathy draw on the perception that the empathizer has adopted concern for the goals of the target, thus sharing their appraisal of an outcome, then evaluations of empathy may incorporate children's evaluations of the underlying target goals. If children disapprove of a target's antisocial goal, we would then expect children to negatively evaluate empathizers relative to counter‐empathizers. As empathy continues to indicate the empathizer's concern for the target's goal, we predict that children will still perceive empathy as evidence of a more positive relationship between the empathizer and target, even when the target's goal is antisocial.

Our hypotheses, materials, data collection, and analysis plan are preregistered on OSF: https://osf.io/b6qwg/.

### 4.1 Methods

#### 4.1.1 Participants

Eighty‐seven children, aged 4 to 7 years old, participated at a children's science museum and in local parks in Southern California (*M*_age_ = 6.18 years, SD_age_ = 1.18 years, 45 girls). An additional three children were tested but were excluded from the final analysis due to experimenter error (1) and being outside the designated age range (2).

Caregivers provided informed consent and completed an optional demographic form. Forty‐seven children were identified by their caregivers as White, 16 as Asian, 17 were identified as belonging to two or more races, and 7 caregivers declined to answer. 17 children were identified as Hispanic or Latinx and 3 caregivers declined to answer. Seventy‐three children came from families where at least one caregiver had a college degree or higher, 8 had at least one caregiver who completed some college, 4 had at least one caregiver who had a high school diploma or GED, 1 had no caregiver who had a high school diploma or GED, and 1 caregiver declined to answer. Participants received a small gift (i.e., temporary tattoos, stickers, or toys) as compensation for their participation. This study and its procedure were approved by the university's institutional review board. Data collection occurred between August 2023 and March 2024.

#### 4.1.2 Materials and Procedure

There were six total stories. In addition to the target and emoter, most stories also mentioned a third, unseen character who was only referred to as a “classmate.” Participants were told about the target's goal and their outcome, as well as the emoter's response following the target's action, which was either empathic or counter‐empathic. Based on the results of Study 1, in which younger children stated it was not “okay” for a character to feel sadness even in an empathic context, we dropped the “okayness” test questions and only asked participants to evaluate how nice each emoter is and infer how much the emoter and target like one another. Block order (moral violation first vs. second), story order (empathic first vs. second), test question order (evaluation first vs. second), and the pairing of stories with empathic and counter‐empathic responses were counterbalanced across participants.

##### 4.1.2.1 Warm Up

**FIGURE 4:**
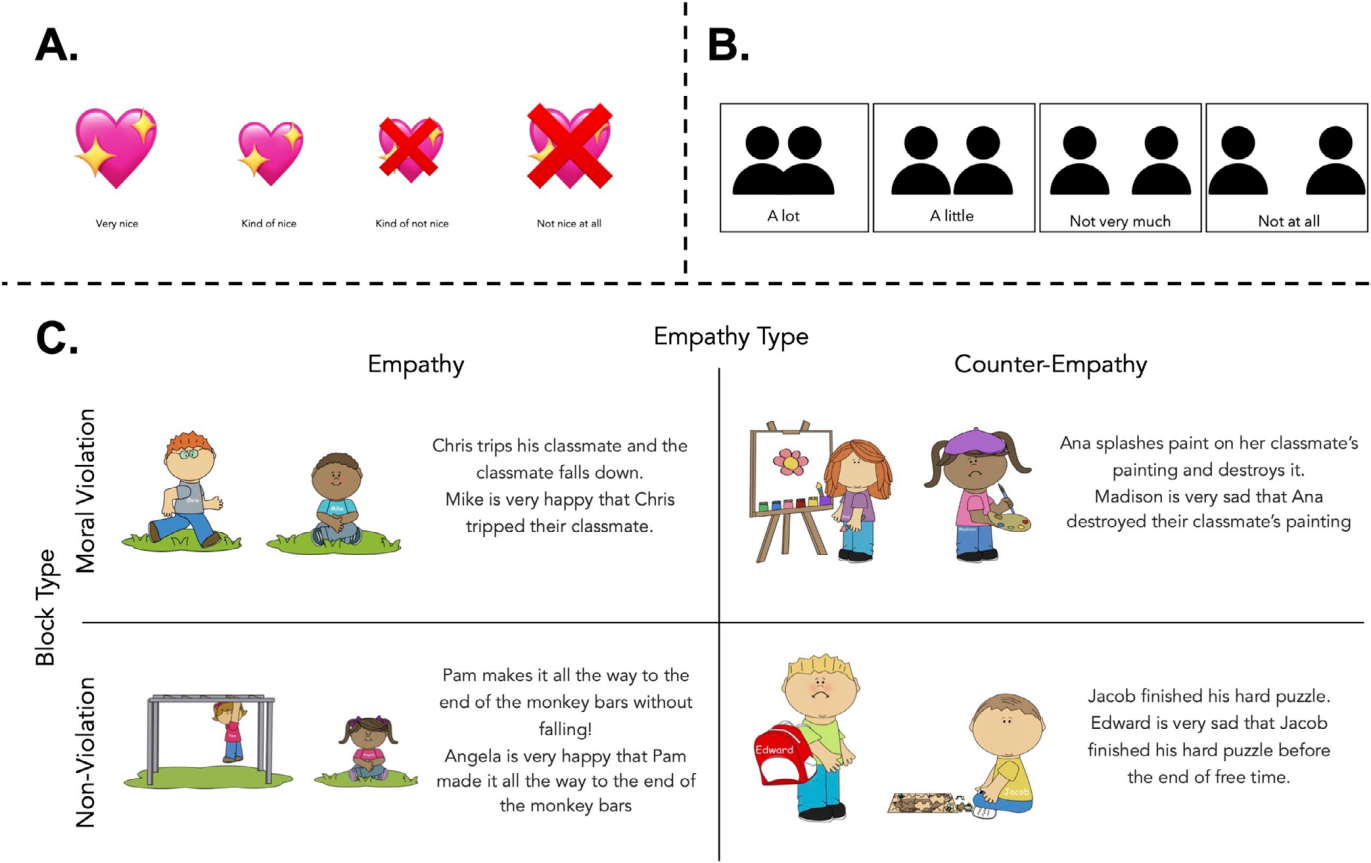
(A) Children were asked to infer how nice the emoter is using a 4‐point scale ranging from “very nice” to “not nice at all”. (B) Children were asked to infer how much the emoter and the actor like each other using a 4‐point scale ranging from “a lot” to “not at all”. (C) Example stimuli from the Moral Violation Block and the Non‐Violation Block.

The researcher began by introducing a 4‐point Likert scale, ranging from “very nice” to “not nice at all”, accompanied by plain or crossed‐out sparkling pink hearts depicting each degree of niceness (Figure 4A). The researcher asked participants how nice it would be if they were given a lot of stickers, and how nice it would be if all of their stickers were stolen. Participants then rated, verbally or by pointing to the scale, how nice it would be if they were given stickers or had them stolen. The researcher then introduced a second 4‐point Likert scale, used to ask how much two people like each other and ranging from “a lot” to “not at all,” accompanied by pictures of two people close together or increasingly far apart (Figure 4B). To familiarize participants with this affiliation scale, we asked participants about their favorite and least favorite characters. Participants rated, verbally or by pointing to the scale, how much they liked their favorite or least favorite character. As preregistered, participants who answered the same regarding their favorite and least favorite characters, and stealing vs. giving stickers, were excluded from the final analysis. No participants were excluded for this reason.

##### 4.1.2.2 Moral Violation Block

In each of four stories, participants were told about a target character and an immoral action they want to commit (e.g., “Ana wants to have the best painting, so Ana is going to destroy her classmate's painting.”) (Figure 4C). The researcher then asked the participant if this was acceptable (e.g., “Is it okay or not okay that Ana is going to destroy her classmate's painting?”). This question served as a manipulation check. Participants who did not answer correctly were excluded from analysis from that story's main analysis. Participants who did not provide an answer were prompted a second time, and were excluded from this story's analysis if they did not provide an answer after the second prompt. All participants answered this initial manipulation check question, but 13 of the 348 total moral violation responses were excluded from the main analysis for indicating the “not okay” actions were “okay”. Following the initial manipulation check, participants then heard the outcome of the action, which was always successful (e.g., “Ana splashes paint on her classmate's painting and destroys it.”). Then, using language structured as in Study 1 and prior work (Smith‐Flores et al. 2023), participants were told about the emoter's vicarious emotion in response to the target's outcome (e.g., “Madison is very happy that Ana destroyed their classmate's painting.”). In two stories, the emoter was described as feeling a congruent, empathic emotion, while in the other two stories the emoter was described as feeling an incongruent, counter‐empathic emotion. This was followed by another manipulation check in which we asked participants to remind us how the emoter feels (e.g., “Remind me, how does Madison feel?”). Following the manipulation check, the researcher restated the emoter's feelings and the situation (e.g., “That's right, Madison is very happy that Ana destroyed their classmate's painting.”). Participants who answered the manipulation check question incorrectly were retold the story once more and asked the manipulation check question again. Four stories were excluded from the final analysis for failing this manipulation check question. Finally, the researcher asked the test questions. Two of the four stories featured an empathizing emoter, and the other two featured a counter‐empathic emoter.

##### 4.1.2.3 Non‐Violation Block

The two stories in the Non‐Violation Block had the same structure as the stories in the Violation Block with the exception of there being no immoral behavior taking place. One story featured an empathic emoter and the other a counter‐empathic emoter. As in the Moral Violation block, we asked participants if the target character's goal (e.g., to cross the monkey bars) was “okay” or “not okay.” Because the target character was not violating a moral rule or norm in these stories, we preregistered exclusion of responses to non‐violation stories from the main analyses when participants said the target character's actions were “not okay.” Thirty‐three of the 174 total non‐violation responses were excluded for this reason. As preregistered, participants who answered both non‐violation “okay” check questions incorrectly were excluded from this block's analysis and replaced, but their responses from the moral violation block were analyzed (*n* = 12). We also included the second manipulation check, in which participants were asked to recall the emoter's response to the target's outcome. No stories in this block were excluded for failing this manipulation check.

##### 4.1.2.4 Dependent Variables

Following each story, participants were asked social evaluation and relationship inference questions. For all test questions, participants who did not provide an answer after an initial prompt were asked a second time before moving on to the next story. All participants who received a second prompt answered and were included in the final analysis.

###### 4.1.2.4.1 Social Evaluation

Participants were instructed to use the niceness scale from the warm‐up questions to evaluate how nice the emoter was in each story (e.g., “How nice is Madison? Very nice, kind of nice, kind of not nice, not nice at all?”).

###### 4.1.2.4.2 Relationship Inference

Participants were also asked to rate the strength of the two characters' relationship (e.g., “How much do Ana and Madison like each other? A lot, a little, not very much, not at all?”).

#### 4.1.3 Description of Analyses

We fit preregistered mixed effects linear regression models of children's responses to all six stories, examining the niceness and closeness ratings separately. Response type (empathy vs. counter‐empathy), block type (moral violation vs. non‐violation), age in years, and the two‐ and three‐way interactions between response type, block type, and age were included in the full model as fixed effects. Response type and block type were dummy coded, and all fixed effects were mean centered. Participant ID was included in the model as a random effect. The resulting full model, applied separately to social evaluations and relationship inferences, was as follows:

$$\begin{array}{c} \text{rating}\sim \text{response type}+\text{block type}+\mathrm{age}+\text{response type}:\\ \text{block type}+\text{response type}:\mathrm{age}+\text{block type}:\mathrm{age}+\text{response type}:\\ \text{block type}:\mathrm{age}+\left(1\;|\;\text{participant}\right). \end{array}$$

Significance of each factor and interaction was assessed using nested model comparisons.

### 4.2 Coding

All responses were coded by the second researcher at the time of the experiment. An additional coder coded 20% of participants from video recordings to ensure reliability. Agreement between coders was 100%.

### 4.3 Results

#### 4.3.1 Social Evaluation

**FIGURE 5:**
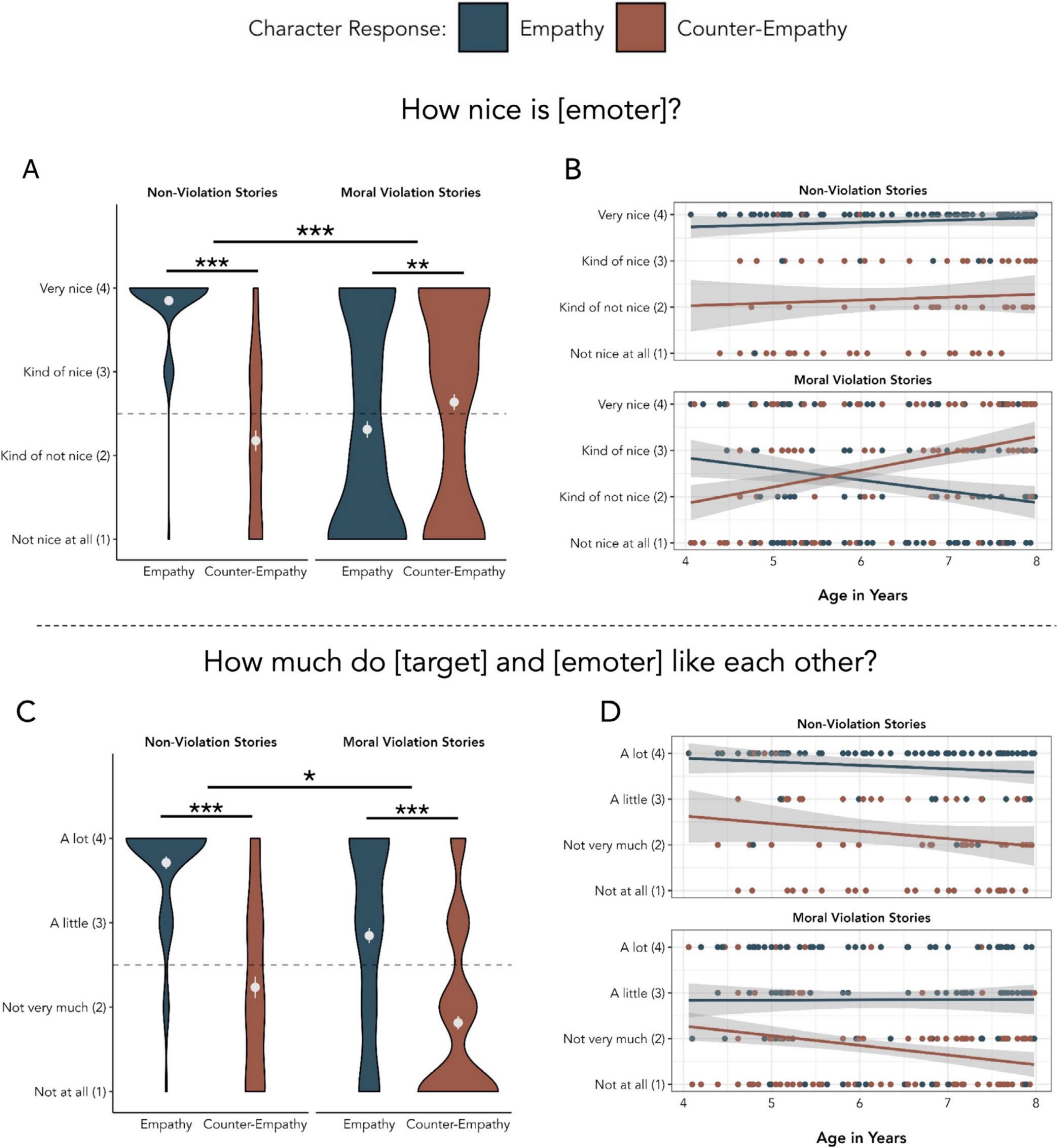
Results from Study 2. (A) Children's social evaluations from Study 2. Means and standard errors are plotted in white. The dashed line represents the mid‐point of the scale (2.5). (B) Children's social evaluations by age. Each dot represents individual children's responses for each story. The gray shaded area represents 95% confidence intervals. (C) Children's relationship inferences from Study 2. (D) Children's relationship inferences by age. **p < 0.01, ***p < 0.001.

Based on the comparison of full and reduced mixed effects models, we observed a main effect of empathy type, χ^2^(1) = 36.580, *p* < 0.001, reflecting higher niceness ratings of empathizers (*M* = 2.785, SEM = 0.085) than counter‐empathizers (*M* = 2.504, SEM = 0.079). This main effect was tempered by a significant interaction between empathy type and block type, χ^2^(1) = 82.924, *p* < 0.001 (Figure 5A). Post hoc pairwise comparisons with Bonferroni adjustments (*α* = 0.0125, 0.05 divided by 4 tests) showed that in the non‐violation block, children rated empathizers as nicer (*M* = 3.849, SEM = 0.054) than counter‐empathizers (*M* = 2.176, SEM = 0.122), *t*(390) = 9.333, *p* < 0.001, 95% CI [1.324, 2.030], *d* = 1.590. In contrast, in the moral violation block children rated counter‐empathizers (*M* = 2.637, SEM = 0.098) as nicer than empathizers (*M* = 2.311, SEM = 0.100), *t*(385) = −3.121, *p* = 0.002, [−0.591, −0.134], *d* = −0.344. Empathizers in the non‐violation block were rated as nicer than empathizers in the moral violation block, *t*(394) = 10.189, *p* < 0.001, [1.231, 1.820], *d* = 1.446, and counter‐empathizers in the moral violation block were rated as nicer than counter‐empathizers in the non‐violation block, *t*(400) = −3.340, *p* = 0.001, [−0.816, −0.211], *d* = −0.487.

There was also a main effect of block type, χ^2^(1) = 21.95, *p* < 0.001—children gave higher niceness evaluations in the non‐violation block (*M* = 3.043, SEM = 0.096) than the moral violation block (*M* = 2.476, SEM = 0.070). There was also a three‐way interaction between response type, block type, and age, ^2^(1) = 10.490, *p* = 0.001. Post hoc comparisons showed an interaction between response type and age in the moral violation block, reflecting increasing niceness ratings for counter‐empathizers and decreasing niceness ratings for empathizers with age, estimate_diff_ = −0.608, *t*(386) = −6.121, *p* < 0.001, 95% CI [−0.803, −0.441], *d* = −0.576 (see Figure 5B). No such response type × age interaction was observed in the non‐violation block, estimate_diff_ = −0.009, *t*(391) = −0.057, *p* = 0.955, 95% CI [−0.316, 0.298], *d* = −0.008.

#### 4.3.2 Relationship Inference

Nested model comparisons showed a main effect of response type, χ^2^(1) = 164.34, *p* < 0.001—children inferred positive relationships from empathy (*M* = 3.114, SEM = 0.071) and negative relationships from counter‐empathy (*M* = 1.936, SEM = 0.067) (Figure 5C). There was also a main effect of block type, χ^2^(1) = 47.462, *p* < 0.001—children inferred more positive relationships in the non‐violation block (*M* = 3.000, SEM = 0.095) than the moral violation block (*M* = 2.325, SEM = 0.066). There was a significant interaction between response type and block type, χ^2^(1) = 6.18, *p* = 0.013. Post hoc comparisons showed children inferred more positive relationships from empathy than counter‐empathy in both the non‐violation block (empathy: *M* = 3.70, SEM = 0.118; counter‐empathy: *M* = 2.22, SEM = 0.122), *t*(386) = 9.854, *p* < 0.001, 95% CI [1.193, 1.78], and in the moral violation block (empathy: *M* = 2.85, SEM = 0.088; counter‐empathy: *M* = 1.81, SEM = 0.088), *t*(383) = 10.856, *p* < 0.001, 95% CI [0.857, 1.24], but the difference in inferred relationship strength was somewhat larger in the non‐violation block. There were no significant main effects or interactions involving age (all *p*s > 0.07) (Figure 5D).

### 4.4 Discussion

As in Study 1, we found that children perceive those who empathize with others' goals as both nicer and more likely to be friends with the target of empathy, so long as the target's goal is morally benign. However, when empathy and counter‐empathy were directed toward immoral actors, children's evaluations reversed: they perceived counter‐empathizers as nicer than empathizers. The strength of this judgment pattern increased with age across our sample of 4‐ to 7‐year‐old children. Relationship inferences did not show the same reversal, as children still inferred that the immoral actors were closer to an observer who empathized with them than one who did not. Relationship inferences showed no evidence of change with age.

Overall, these results are consistent with the hypothesis that children do not just heuristically approve of empathizers, but rather take into account what empathy and counter‐empathy imply about what the emoter values. This pattern was not as evident among the 4‐year‐old participants in our sample, who were more likely to judge empathizers and counter‐empathizers of immoral acts as nice and not nice, respectively. Future research should investigate whether the basis for children's evaluations of empathy changes with age, from straightforward approval of any empathy to a consideration of underlying goals, or whether the difference in younger children's reasoning merely reflected performance limitations. Another topic for future investigation is the extent to which children attribute vicarious emotions expressed in the context of moral violations as directed toward the transgressor versus the victim of the transgression. It is unlikely that participants in the current experiment perceived the emoter's feelings as primarily victim‐oriented, as the emotions were stated to be about the target, there was only a specific victim present in one of the scenarios, and participants made inferences from the emotions about the emoter‐target relationship. However, in other scenarios, children may make different evaluations of people who respond more to the victim vs. the transgressor.

## 5 General Discussion

Across two samples of 4‐ to 7‐year‐old children, we found that children typically evaluate empathizers positively and counter‐empathizers negatively. This pattern was apparent in judgments of both appropriateness and niceness; it held for vicarious emotional responses to others' positive and negative outcomes, and it occurred despite no discussion of overt expressions or actions (i.e., comforting or mocking) toward the subject of the vicarious emotion. These results are consistent with previous findings that young children positively or negatively evaluate puppets who comfort or mock a person in distress (Geraci et al. 2021; Paulus et al. 2020), but also go beyond them, showing that children use information about people's internal feelings toward others' welfare to guide judgments in both positive and negative situations.

We also replicated and extended the finding that children use vicarious emotions to infer social relationships (Smith‐Flores et al. 2023). As in the previous work, children inferred that empathizers were closer than counter‐empathizers to the target of their vicarious emotions. Here, in addition, we found that children used empathy and counter‐empathy to infer closer or more distant relationships, respectively, than targets had with neutral responders. Thus, both types of vicarious emotions provide information about the nature of interpersonal ties.

When we contrasted empathy with helping behavior in Study 1, both evaluations of empathy and inferences about relationships changed. Children thought helping without empathizing was nicer and more appropriate than empathizing without helping. They also thought a recipient of those two response types was more likely to be friends with the helper than the empathizer. These results provide evidence that children initially weigh helping more heavily than empathy in their social evaluations. This is consistent with the finding that young children across a number of cultures consider helping to be obligatory, regardless of the relationship between the actor and recipient (Marshall et al. 2022). There may be other scenarios that would have elicited different judgments, however, including ones in which empathy is perceived to provide comfort or actions aimed at helping are relatively ineffective. We also found that children's appropriateness ratings changed with age, such that older children rated the helpful and empathic responses more similarly than younger children did. This is consistent with work finding that older children and adults place considerable weight on vicarious emotion in their social evaluations. For instance, older children rate comforting those in distress as both desirable and obligatory (Tavassoli et al. 2023), while adults consider helpful and compassionate traits as similarly important for judging moral character (Landy and Perry 2024). Future research should also investigate whether, like adults, children's evaluations are sensitive to the costliness of empathy and helping in different situations (Andreoni et al. 2017; Cameron et al. 2016, 2019).

When we manipulated the moral status of the target of vicarious emotion in Study 2, a different pattern emerged. Children no longer positively evaluated story characters who empathized with immoral actors and instead rated counter‐empathizers as nicer. They did, however, continue to infer that the empathizer was closer friends with the target than the non‐empathizer. The former result indicates that children do not base their evaluations on a simple heuristic that all empathy is prosocial, while the latter demonstrates that children do not need to perceive empathy as nice in order to view it as evidence of a friendship. Instead, like adults, children's evaluations of empathy and counter‐empathy depend on the target of those vicarious responses, even though empathy is consistently viewed as evidence of interpersonal concern and social affiliation (Wang and Todd 2021).

The overall pattern of results is consistent with the hypothesis that children reason about vicarious emotions within the framework of a broader intuitive psychology. In standard accounts of intuitive psychology, children and adults use information about beliefs and desires to predict the actions a person will take, as well as the emotions the person will feel based on their appraisals of situations and outcomes (Jara‐Ettinger et al. 2016; Ong et al. 2019; Wellman et al. 2000). To incorporate affiliation, this intuitive theory can be expanded to posit that people adopt concern for the rewards and welfare of social partners they care about, either due to a specific relationship or a broadly prosocial disposition (Jara‐Ettinger et al. 2016; Kleiman‐Weiner et al. 2017; Powell 2022). This can include desiring negative outcomes for social partners that a person dislikes or is in competition with. Positive or negative concern for another person's welfare can then be expected to lead, via appraisals of their experiences, to vicarious emotions (Smith‐Flores and Powell 2023; Wondra and Ellsworth 2015). When users of this expanded intuitive psychology see one person have a good or bad experience, they should thus expect observers who care about the person to feel empathic happiness or sadness, respectively. Negative affiliation with the experiencer would be expected to lead to counter‐empathic emotions, instead. In addition to allowing observers to predict vicarious emotions, based on what they know about a person's relationships or moral character and the person's knowledge of others' well‐being, this sort of intuitive theory can also support reverse inference, allowing observers who use it to make inferences about relationships or character from observations of vicarious emotion (Gerstenberg and Tenenbaum 2017).

This account predicts the current findings well. It explains why children typically positively evaluate empathizers and negatively evaluate counter‐empathizers: empathy provides evidence of prosocial concern for others' welfare, while counter‐empathy indicates a desire for others to fail or suffer. It also explains why presenting empathy or counter‐empathy toward an immoral goal changes evaluations but not relationship inferences: children still represent the empathizer or counter‐empathizer as sharing or disputing the target's goal, thus indicating the valence of the relationship between the two, but they also represent the empathizer and counter‐empathizer as endorsing or denigrating the immoral goal themselves, impacting inferences about the emoters' character. Finally, this framework could provide insight into children's emphasis on helping over empathizing: empathy may be taken as false or shallow evidence for interpersonal concern if that concern does not also motivate effortful helping.

A number of age effects in the current findings suggest that children's use of this intuitive psychological framework strengthens with age. The youngest participants frequently failed to endorse empathic sadness as okay, and were also somewhat less likely to use this response to infer positive relationships (see Data S1). The impact of immoral goals on children's evaluations of empathy also strengthened with age, suggesting that younger children may rely more on the heuristic that all empathy is prosocial, while older children more fully consider what empathy with a given act implies about the empathizer's values. Finally, age‐related changes in children's responses to conflicting cues of helping and empathy may also reflect a more nuanced intuitive psychology. Despite philosophical concerns that emotional motivations for helping undermine human altruism, empathic concern tends to enhance adults' lay evaluations of helpers' moral character. Consistent with our proposed intuitive psychological framework, this reflects the inference that emotional helpers are genuinely concerned about others, while unemotional helpers may be motivated by reputational or reciprocity‐based rewards for themselves (Barasch et al. 2014). We found that older children were somewhat less approving of helpful but counter‐empathic responses, potentially reflecting the onset of similar reasoning.

Participants in the current research and other recent research on children's evaluations of empathy and comforting (Geraci et al. 2021; Paulus et al. 2020) are from industrialized, Western cultures and families with higher than average parent education levels. The prevalence of empathy does vary measurably across populations (Chopik et al. 2017; Piff and Robinson 2017), and this may be related to variability in the strength of children's empathy‐based social evaluation. However, there are also reasons to expect that similar patterns would be observed in other populations. For example, intentions play some role in moral reasoning across many populations (Barrett and Saxe 2021), and so may emotions that provide information about underlying social motives. Future research should investigate these possibilities.

In sum, these results are broadly consistent with research showing that children's moral reasoning is sensitive to the intentions behind actions, not just action outcomes (Cushman et al. 2013; Killen et al. 2011; Woo et al. 2022). Both that research and the current findings indicate that children, like adults, attend not just to the way a person acts toward others, but also to information about the person's underlying mental states, which can in turn support inferences about the person's moral motives or character (Barasch et al. 2014; Carlson et al. 2022; Goodwin et al. 2014). Future research should examine how this understanding of empathy impacts children's participation in social interactions, including both their choice of social partners and their use of expressions of empathy to communicate their own social characteristics.

## Author Contributions

**Alexis S. Smith‐Flores:** conceptualization, methodology, formal analysis, visualization, writing – original draft, writing – review and editing. **Gabriel J. Bonamy:** investigation, methodology, writing – original draft. **Lindsey J. Powell:** conceptualization, funding acquisition, methodology, formal analysis, supervision, writing – review and editing.

## Ethics Statement

This study was conducted with approval from the UC San Diego Institutional Review Board.

## Conflicts of Interest

The authors declare no conflicts of interest.

## Supporting information


Data S1.

## Data Availability

Both studies were preregistered before data collection began. The research plan (including initial hypotheses and the analysis plan), materials and stimuli, data, and code used in the analysis are publicly available on OSF: https://osf.io/b6qwg
