# Supplementary material for: Children's Evaluations of Empathizers
Source: Child Dev. 2025 Apr 4;96(4):1290–305. doi: 10.1111/cdev.14242 (PMC12208016; doi:10.1111/cdev.14242)
Supplement: Supplementary file 1 — Data S1. [file CDEV-96-1290-s001.docx]

**Supplemental Material**

**Study 1**

*Exploratory Non-Preregistered Analyses – Binary Logistic Regressions for Social Evaluation and Relationship Inference Questions*

Given the age-related change in children’s appropriateness evaluations, we conducted exploratory nested logistic regression model comparisons to test whether age had an effect on children’s responses in Study 1.

**Empathy vs. Counter-Empathy Block.** In children’s social evaluation of whether the empathizer or the counter-empathizer was nicer, there was a main effect of age, 𝛘^2^ (1) = 8.48, *p* < .004 – children were more likely to select the empathizer as nicer as they got older. In their relationship inferences, there was an interaction between age and outcome type, 𝛘^2^ (1) = 4.402, *p* = .036 – children’s responses in the positive outcome story did not change with age, while their selection of the empathizer increased with age in the negative outcome story.

**Comparison-to-Neutral Block.** There were no age-related differences in either the social evaluation, 𝛘^2^ (1) = 2.111, *p* = .146, or the relationship inference question, 𝛘^2^ (1) = 2.398, *p* = .122. Though, there was a difference between children’s responses across stories in the social evaluation question, 𝛘^2^ (1) = 13.986, *p* < .001 – children selected the empathizer when compared to the neutral character more often than they chose the neutral character compared to the counter-empathizer. This was not the case in the relationship inference question, 𝛘^2^ (1) = 0.814, *p* = .367.

**Helping Block.** There were no age-related differences in either the social evaluation, 𝛘^2^ (1) = 0.848, *p* = .357, or the relationship inference question, 𝛘^2^ (1) = 0.086, *p* = .769.

*Exploratory Non-Preregistered Analyses — Participant Sex*

To examine whether participant sex influenced participants’ initial appropriateness ratings of vicarious emotions, we included the interaction between participant sex and response type in the full model for each block. These models also included participant age given the significant interaction between age and response type across the blocks. We found no significant interaction between sex and response type in any of the blocks: Empathy vs. Counter-Empathy Block (*F*(1, 279) = 0.070, *p* = .791), Comparison-to-Neutral Block (*F*(1, 271) = 0.084, *p* = .772), or Helping Block (*F*(1, 275) = 2.978, *p* = .086).

**Study 2**

*Confirmatory Preregistered Analyses – Individual Block Analyses*

To further examine children’s responses to the social evaluation and relationship inference questions in Study 2, we preregistered individual block analyses for each of the test questions. These additional analyses mirrored the collapsed analyses described in the main text.

**Non-violation Block**. In the social evaluation responses, there was a main effect of empathy, 𝛘^2^ (1) = 110.00, *p* < .001 – children rated empathizers more positively than counter-empathizers, but there was no main effect of age, 𝛘^2^ (1) = 0.978, *p* = .323, or interaction between age and empathy, 𝛘^2^ (1) = 0.012, *p* = .912. Similar results were observed in the relationship inference question. There was a main effect of empathy, 𝛘^2^ (1) = 77.995, *p* < .001 – children inferred more positive relationships from empathy than counter-empathy, but there was no main effect of age, 𝛘^2^ (1) = 3.778, *p* = .052, or interaction between age and empathy, 𝛘^2^ (1) = 0.504, *p* = .478. These results were in line with our preregistered predictions.

**Moral Violation Block**. In the social evaluation responses, there was a main effect of empathy, 𝛘^2^ (1) = 8.323, *p* = .004 – children rated counter-empathizers more positively than empathizers. There was no main effect of age, 𝛘^2^ (1) = 0.700, *p* = .403, but there was an interaction between age and empathy, 𝛘^2^ (1) = 29.596, *p* < .001. A post-hoc pairwise comparison showed that as children got older they were more likely to positively evaluate counter-empathy and negatively evaluate empathy in response to moral violations, estimate_diff_ = -0.605, t(199) = -5.581, p < .001, 95% CI [-0.819, -0.392], *d* = -0.526.

In the relationship inference question, there was a main effect of empathy, 𝛘^2^ (1) = 90.135, *p* < .001 – children inferred more positive relationships from empathy than counter-empathy. There was no main effect of age, 𝛘^2^ (1) = 2.905, *p* = .088, but there was an interaction between age and empathy, 𝛘^2^ (1) = 5.302, *p* = .021. A post-hoc pairwise comparison showed that as children got older they were more likely to infer a negative relationship from counter-empathy, estimate_diff_= 0.198, t(157) = 2.300, p = .023, 95% CI [0.029, 0.368], *d* = 0.217.

*Exploratory Preregistered Analyses* – *Order Effects*

Across participants in Study 2, empathy type order (empathy first vs. second) and block order (moral violation block first vs. second) is counterbalanced. To examine any effect order may have had on children’s responses we will conduct the confirmatory mixed-effect linear regressions again but include empathy type order and block order in the models as dummy-coded, mean-centered fixed effects. We used a nested model comparison to determine whether the addition of fixed order effects improved the model’s fit. We found no empathy order effect, 𝛘^2^ (1) = 0.249, *p* = .618, or block order effect, 𝛘^2^ (1) = 0.645, *p* = .422, on the social evaluation question. We also found no empathy order effect, 𝛘^2^ (1) = 0.013, *p* = .910, on the relationship inference question. There was a block order effect on the relationship inference question, 𝛘^2^ (1) = 5.240, *p* = .022 – children’s responses were more positive overall when they heard the non-violation block first.

*Exploratory Non-Preregistered Analyses — Participant Sex*

To examine whether participant sex influenced their niceness evaluations and social relationship inferences, we included the interaction between participant sex and response type in the full model for each block. We found no significant interaction between sex and response type in either of the blocks: Moral Violation Block (𝛘^2^ (1) = 0.225, *p* = .635) and Non-Violation Block (𝛘^2^ (1) = 1.973, *p* = .160).
